# *XRCC3* Thr241Met and *TYMS* variable number tandem repeat polymorphisms are associated with time-to-metastasis in colorectal cancer

**DOI:** 10.1371/journal.pone.0192316

**Published:** 2018-02-02

**Authors:** Yanjing He, Michelle E. Penney, Amit A. Negandhi, Patrick S. Parfrey, Sevtap Savas, Yildiz E. Yilmaz

**Affiliations:** 1 Department of Mathematics and Statistics, Faculty of Science, Memorial University of Newfoundland, St. John's, Canada; 2 Discipline of Genetics, Faculty of Medicine, Memorial University of Newfoundland, St. John’s, Canada; 3 Discipline of Medicine, Faculty of Medicine, Memorial University of Newfoundland, St. John’s, Canada; 4 Discipline of Oncology, Faculty of Medicine, Memorial University of Newfoundland, St. John’s, Canada; Istituto di Ricovero e Cura a Carattere Scientifico Centro di Riferimento Oncologico della Basilicata, ITALY

## Abstract

**Background:**

Metastasis is a major cause of mortality in cancer. Identifying prognostic factors that distinguish patients who will experience metastasis in the short-term and those that will be free of metastasis in the long-term is of particular interest in current medical research. The objective of this study was to examine if select genetic polymorphisms can differentiate colorectal cancer patients based on timing and long-term risk of metastasis.

**Methods:**

The patient cohort consisted of 402 stage I-III colorectal cancer patients with microsatellite instability (MSI)-low (MSI-L) or microsatellite stable (MSS) tumors. We applied multivariable mixture cure model, which is the proper model when there is a substantial group of patients who remain free of metastasis in the long-term, to 26 polymorphisms. Time-dependent receiver operator characteristic (ROC) curve analysis was performed to determine the change in discriminatory accuracy of the models when the significant SNPs were included.

**Results:**

After adjusting for significant baseline characteristics, two polymorphisms were significantly associated with time-to-metastasis: TT and TC genotypes of the *XRCC3* Thr241Met (p = 0.042) and the 3R/3R genotype of *TYMS* 5’-UTR variable number tandem repeat (VNTR) (p = 0.009) were associated with decreased time-to-metastasis. ROC curves showed that the discriminatory accuracy of the model is increased slightly when these polymorphisms were added to the significant baseline characteristics.

**Conclusions:**

Our results indicate *XRCC3* Thr241Met and *TYMS* 5’-UTR VNTR polymorphisms are associated with time-to-metastasis, and may have potential biological roles in expediting the metastatic process. Once replicated, these associations could contribute to the development of precision medicine for colorectal cancer patients.

## Introduction

Colorectal cancer is a worldwide health concern, especially in developed regions [1]. It is expected to cause 8.3% of all cancer deaths in the United States [2] and 12% of all cancer deaths in Canada in 2016 [3]. In North America the current estimated 5-year survival for this disease is 63–65% [2,3]. However, this rate reduces to a striking 13% in patients whose cancer has metastasized [2]. Consequently, it is a critical aim in healthcare to differentiate between patients who will experience metastasis within a short time and those who will be free of metastasis in the long-term.

When considering metastasis as the disease outcome, it is possible that a proportion of the cohort does not experience the outcome during a long follow up period. Thus, such cohorts consist of patients who experience metastasis (i.e. patients who are “susceptible” to metastasis) as well as those that remain free of metastasis in the long term (i.e. patients who are “statistically cured” or “long-term metastasis-free survivors”) [4–7]. When the population is a combination of such patients, an advanced statistical approach known as the mixture cure model is appropriate in modeling time to metastasis. While rarely used, this model can provide novel insight into cancer prognosis [7–9]. This model serves two purposes: a) it can identify prognostic factors which are able to differentiate between patients who are susceptible to develop metastasis and who will potentially remain metastasis-free in the long-term, and b) within the susceptible patient sub-group, this model can identify prognostic factors associated with time-to-metastasis. In colorectal cancer, it is well known that the tumor MSI-high (MSI-H) phenotype is associated with substantially reduced risk of metastasis [10,11]. In contrast, patients with MSI-L or MSS tumor phenotypes are a mixture of two subgroups: patients who are the long-term metastasis-free survivors and patients susceptible to metastasis. This suggests the existence of other factors that can influence the long-term risk and timing of metastasis in patients with the MSI-L/MSS subtype of colorectal cancer. To identify such factors, we applied the mixture cure model to 26 genetic polymorphisms in 402 colorectal cancer patients with the MSI-L/MSS tumor phenotype.

## Materials and methods

### Ethics statement

Patient consent was obtained by Newfoundland Colorectal Cancer Registry (NFCCR) [12–14] at the time of recruitment. If the patient was deceased, consent was sought from a close relative [12]. Ethics approval for this study was obtained from the Health Research Ethics Board (HREB, #09.106), Ethics Office, Health Research Ethics Authority, St. John’s, NL, Canada.

### Polymorphisms, genotype data, and patient cohort

We examined the 26 polymorphisms listed in Table 1. These polymorphisms were previously selected, genotyped, and investigated as a part of another survival study, details of which can be found in Negandhi et al. [15]. Briefly, polymorphisms were selected for inclusion into the study if the polymorphism was found to be significantly associated with overall survival time in colorectal cancer in at least one study till August 2010 and was able to be genotyped by the methods used by the authors [15]. While the data from 532 patients was available in the Negandhi et al. [15] study, in order to address our research question, in the present study patients with stage IV tumors were excluded because they already had metastatic cancer at diagnosis. In addition, patients with MSI-H tumors were excluded since patients with MSI-H tumor phenotype are very unlikely to experience metastasis [10,11]. Thus, our study cohort consisted of 402 stage I-III patients with MSI-L/MSS tumors. Of these patients, 21% experienced metastasis after diagnosis. The median follow-up time for metastasis in this cohort was 6.3 years and the maximum follow-up time was 10.9 years. Of note, there were no stage I-III patients with MSI-H tumors who experienced metastasis in the patient cohort (S1 Fig). Also note that there were three patients with 4R allele in *TYMS* rs34743033 in our study cohort; this allele was treated like 3R allele during the analysis.

**Table 1:** Candidate polymorphisms investigated in the patient cohort.

Gene [16]	Genomic Location [17,18]*	rs Number	Polymorphism	Minor allele and MAF
*GSTT1*	Chr22: 24,376,133–24,384,680	NA	gene deletion	deletion allele, 17.2%
*GSTM1*	Chr1: 110,230,436–110,251,661	NA	gene deletion	deletion allele, 45.5%
*ERCC2*	Chr 19, 45854919	rs13181	Lys751Gln, G/T	G, 34.2%
*GSTP1*	Chr 11, 67352689	rs1695	Ile105Val, A/G	G, 37.4%
*MTHFR*	Chr 1, 11854476	rs1801131	Glu429Ala, A/C	C, 31.9%
*MTHFR*	Chr 1, 11856378	rs1801133	Ala222Val, C/T	T, 30.9%
*VEGFA*	Chr 6, 43738350	rs2010963	-634G/C in 5'-UTR	C, 26.2%
*XRCC1*	Chr 19, 44055726	rs25487	Arg399Gln, G/A	A, 35.4%
*ERCC5*	Chr 13, 103504517	rs1047768	His46His, C/T	T, 40.2%
*OGG1*	Chr 3, 9798773	rs1052133	Ser326Cys, C/G	G, 21.8%
*ERCC1*	Chr 19, 45923653	rs11615	Asn118Asn, C/T	C, 36.4%
*TYMS*	Chr 18, 673444	NA**	indel 6 bp in 3'-UTR	del, 33.2%
*MLH1*	Chr 3, 37053568	rs1799977	Ile219Val, A/G	G, 29.7%
*FAS*	Chr 10, 90749963	rs1800682	c.-24+733T >C	C, 44.3%
*IL6*	Chr 7, 22766645	rs1800795	-174G/C in promoter	C, 41.8%
*EGFR*	Chr 7, 55229255	rs2227983	Arg521Lys, G/A	A, 26.9%
*DCC*	Chr 18, 50432602	rs2229080	Arg201Gly, C/G	G, 37.8%
*MMP2*	Chr 16, 55511806	rs243865	-1306C/T in promoter	T, 22.9%
*VEGFA*	Chr 6, 43752536	rs3025039	+936C/T in 3'-UTR	T, 9.8%
*FGFR4*	Chr 5, 176520243	rs351855	Gly388Arg, A/G	T, 31.5%
*XRCC3*	Chr 14, 104165753	rs861539	Thr241Met, C/T	T, 39.9%
*CCND1*	Chr 11, 69462910	rs9344	Pro241Pro, A/G	A, 44.6%
*EXO1*	Chr 1, 242048674	rs9350	Pro757Leu, C/T	T, 14.2%
*SERPINE1*	Chr 7, 100769711	rs1799889	-675 indelG in promoter	G, 47.7%
*MMP1*	Chr 11, 102670496	rs1799750	-1607 indel G in promoter	G, 45.6%
*TYMS*	Chr 18; 657,396–657,980	rs34743033	2R/3R in 5'-UTR	2R, 45.8%

*Genomic location from GRCh37.p13 assembly of the polymorphism according to dbSNP for the SNPs and Ensembl database for the gene deletions.

**This polymorphism was previously known as rs16430.

MAF: minor allele frequency, NA: not available.

### Data analysis

The survival outcome of interest was time-to-metastasis. Patients who did not experience metastasis by the end of the follow-up time were censored at the time of the last follow-up.

We modeled time-to-metastasis by using the logistic Weibull cure model, a parametric proportional hazard mixture cure model [7,19], which is referred as the mixture cure model. By fitting this model, we could measure and test associations between the genetic polymorphisms and (i) the long-term risk of experiencing metastasis, as well as (ii) the time-to-metastasis in patients who are susceptible to metastasis after their diagnosis. Specifically, this model provides inferences for both components of the disease outcome: odds ratio estimate for the long-term risk of experiencing metastasis and hazard ratio estimate for the time-to-metastasis in patients who are susceptible to metastasis. In the estimation method, it is assumed that the censored survival times follow the same survival probability pattern [20]. Therefore, we use the term “patients who are susceptible to metastasis” to define the patients who experienced or will eventually experience metastasis.

Prior to analysis, Kaplan-Meier survival curves were obtained for the baseline characteristics (S2 Fig) and polymorphisms to verify that the mixture cure model was the appropriate model. These curves were used to determine (and eventually confirmed) if, for each category of a given variable, we had mixtures of long-term metastasis-free patients and patients susceptible to metastasis. This can be visually assessed if each curve reaches a plateau at a non-zero survival probability [7] (i.e. beyond a time-point there was no metastasis and patients’ time-to-metastasis were censored).

As the first step in conducting multivariable analysis using the mixture cure model, we needed to identify baseline characteristics that were significantly associated with the long-term risk or timing of metastasis in our patients. To do this, we used the purposeful selection of covariates method described by Hosmer et al. [21]. First, all the available baseline characteristics (Table 2) significantly associated with the long-term risk or timing of metastasis in a univariable analysis at a p-value threshold of 0.2 were included in the initial multivariable model. After fitting this initial model, a backward selection method was applied to obtain the final model while considering association tests with each variable originally not selected. If a variable originally not in the initial model was significantly associated with long-term risk or timing of metastasis in the multivariable model under consideration, then it was included in the model. In addition, if a baseline characteristic was insignificant in the model but its removal caused a significant change in at least one of the coefficient estimates of another baseline characteristic, it was put back into the model. The final significant baseline characteristics were tumor location (rectum versus colon), tumor grade (poorly differentiated/undifferentiated versus well differentiated/moderately differentiated), *BRAF* V600E mutation status (present versus absent), adjuvant 5-fluorouracil (5-FU) chemotherapy treatment status (yes versus no), and stage (II versus I and III versus I) (S1 Table and S2 Fig). Then, each polymorphism was examined in multivariable mixture cure models adjusting for these significant baseline characteristics. In this study, the dominant genetic model was applied. To implement this model, patients with minor allele homozygous genotype were combined with the heterozygous patients. This patient group then was compared to patients who were homozygous for the major allele.

**Table 2:** Baseline characteristics and metastasis distribution in the patient cohort.

Variable		Total	Number of Metastasis*	% of Metastasis
Sex	Female	145	30	20.7%
	Male	257	56	21.8%
Age	(21–60]	167	44	26.3%
	(60–70]	163	31	19.0%
	>70	72	11	15.3%
Familial risk	Low	203	35	17.2%
	Intermediate/high	199	51	25.6%
5-FU based treatment	5-FU treated	228	63	27.6%
	Other/no chemo	168	18	10.7%
	Unknown	6	5	83.3%
Stage	I	84	8	9.5%
	II	167	32	19.2%
	III	151	46	30.5%
Location	Colon	248	42	16.9%
	Rectum	154	44	28.6%
Histology	Non-mucinous	363	78	21.5%
	Mucinous	39	8	20.5%
Grade	Well/moderately diff.	376	84	22.3%
	Poorly diff./undiff.	23	2	8.7%
	Unknown	3	0	0.0%
Vascular Invasion	Absent	256	47	18.4%
	Present	118	31	26.3%
	Unknown	28	8	28.6%
Lymphatic Invasion	Absent	250	46	18.4%
	Present	123	32	26.0%
	Unknown	29	8	27.6%
*BRAF* Val600Glu mutation	Absent	355	76	21.4%
	Present	20	9	45.0%
	Unknown	27	1	3.7%

*There may be an underestimation in the frequency of metastasis due to right censoring (median follow-up time = 6.3 years).

diff.: differentiated, 5-FU: 5-fluorouracil.

A p-value less than 0.05 was considered significant unless otherwise was stated.

To determine if the polymorphisms can more accurately discriminate between patients in terms of their risk of metastasis at different time points, we performed time-dependent receiving operator characteristic (ROC) analysis [22]. The area under the ROC curve (AUC) for different models including the adjustment factors and/or significant polymorphisms provides a numeric reference for assessing the discriminatory accuracy of the models.

All statistical analyses were performed using R v. 3.0.1.

## Results

The baseline characteristics for this cohort can be found in Table 2. One-fifth (n = 86) of the patients in the cohort experienced metastasis within the follow-up time. A higher proportion of stage III patients experienced metastasis (31%) compared to stages I (10%) and II (19%). As expected, there were more men (64%) than women (36%) in the patient cohort, but the frequency of metastasis was similar for both sexes (~21%). Also, there were substantially more patients with non-mucinous (90%) compared to mucinous (10%) tumors, but the frequency of metastasis for each group was similar (approximately 21%). Approximately half (57%) the patients were treated with 5-FU chemotherapy and, of those, 28% experienced metastasis. Most patients had well- or moderately-differentiated tumors (94%) and a higher proportion had tumors located in the colon (62%) compared to the rectum (38%).

Mixture cure model provided two association test results for each polymorphism: one for the association with the long-term metastasis risk (in all patients) and the second with time-to-metastasis (in patients who are susceptible to metastasis). In our study, after adjusting for significant baseline characteristics, two out of 26 polymorphisms showed significant associations with time-to-metastasis in patients who are susceptible to metastasis. The genotypes containing the minor allele (TT + TC) of *XRCC3* rs861539 (HR = 2.06, p = 0.042) and genotypes homozygous for 3 repeats (3R/3R) in *TYMS* rs34743033 (HR = 0.40, p = 0.009) were significantly associated with a shorter time-to-metastasis in patients who were susceptible to metastasis after diagnosis (Tables 3 and 4). On the other hand, none of the polymorphisms exhibited significant associations with the long-term risk of metastasis (S2 Table).

**Table 3:** Multivariable mixture cure model results for *XRCC3* rs861539 after adjusting for significant baseline characteristics.

	Time-to-metastasis among susceptible patients			Long-term risk of metastasis		
Variable (*a* vs. *b*)	HR	95% CI	p-value	OR	95% CI	p-value
Location (Rectum vs. Colon)	0.31	(0.14, 0.69)	0.004	6.91	(1.89, 25.20)	0.003
Grade (poorly diff./undiff. vs. well/moderately diff.)	7.25	(0.64, 82.11)	0.110	0.05	(0.01, 0.51)	0.012
*BRAF* Mutation Status (Present vs. Absent)	1.53	(0.62, 3.80)	0.357	4.10	(1.27, 13.24)	0.018
5-FU Treatment (Yes vs. No)	0.26	(0.08, 0.82)	0.021	4.21	(0.87, 20.52)	0.075
Stage II vs. Stage I	2.16	(0.32, 14.39)	0.427	1.81	(0.32, 10.31)	0.505
Stage III vs. Stage I	10.24	(1.19, 88.51)	0.034	0.93	(0.08, 10.27)	0.953
*XRCC3* rs861539 (TT+TC vs. CC)	2.06	(1.03, 4.13)	0.042	1.05	(0.46, 2.40)	0.911

Multivariable mixture cure model included *XRCC3* rs861539 and significant baseline characteristics identified in this study (n = 366 patients): tumor location, histologic grade, *BRAF* mutation status, 5-FU treatment status, and disease stage.

HR: hazard ratio for time-to-metastasis among susceptible group. HR compares metastasis rate in subgroup *a* with that in subgroup *b* among those who are susceptible to metastasis.

OR: odds ratio for metastasis (i.e., probability of being in susceptible group). OR compares metastasis proportion in subgroup *a* with that in subgroup *b*.

CI: confidence interval, diff.: differentiated, 5-FU: 5-fluorouracil.

**Table 4:** Multivariable mixture cure model results for *TYMS* rs34743033 after adjusting for significant baseline characteristics.

	Time-to-metastasis among susceptible patients			Long-term risk of metastasis		
Variable (*a* vs. *b*)	HR	95% CI	p-value	OR	95% CI	p-value
Location (Rectum vs. Colon)	0.38	(0.16, 0.87)	0.022	8.11	(2.00, 32.89)	0.003
Grade (poorly diff./undiff. vs. well/moderately diff.)	10.42	(0.92, 117.70)	0.058	0.04	(0.01, 0.42)	0.008
*BRAF* Mutation Status (Present vs. Absent)	1.65	(0.57, 4.76)	0.355	4.84	(1.36, 17.19)	0.015
5-FU Treatment (Yes vs. No)	0.30	(0.09, 0.95)	0.040	4.99	(0.93, 26.64)	0.060
Stage II vs. Stage I	3.42	(0.26, 45.43)	0.352	0.97	(0.06, 16.28)	0.983
Stage III vs. Stage I	17.49	(1.18, 258.50)	0.037	0.38	(0.01, 12.69)	0.589
*TYMS* rs34743033 (2R/2R +2R/3R vs. 3R/3R*)	0.40	(0.20, 0.80)	0.009	2.41	(0.85, 6.82)	0.096

Multivariable mixture cure model included *TYMS* rs34743033 and significant baseline characteristics identified in this study (n = 366 patients): tumor location, histologic grade, *BRAF* mutation status, 5-FU treatment status, and disease stage.

*There were three patients with a 4R allele of *TYMS* rs34743033; this allele was treated as 3R during the analyses.

HR: hazard ratio for time-to-metastasis among susceptible group. HR compares metastasis rate in subgroup *a* with that in subgroup *b* among those who are susceptible to metastasis.

OR: odds ratio for metastasis (i.e., probability of being in susceptible group). OR compares metastasis proportion in subgroup *a* with that in subgroup *b*.

CI: confidence interval, diff.: differentiated, 5-FU: 5-fluorouracil.

**Fig 1:**
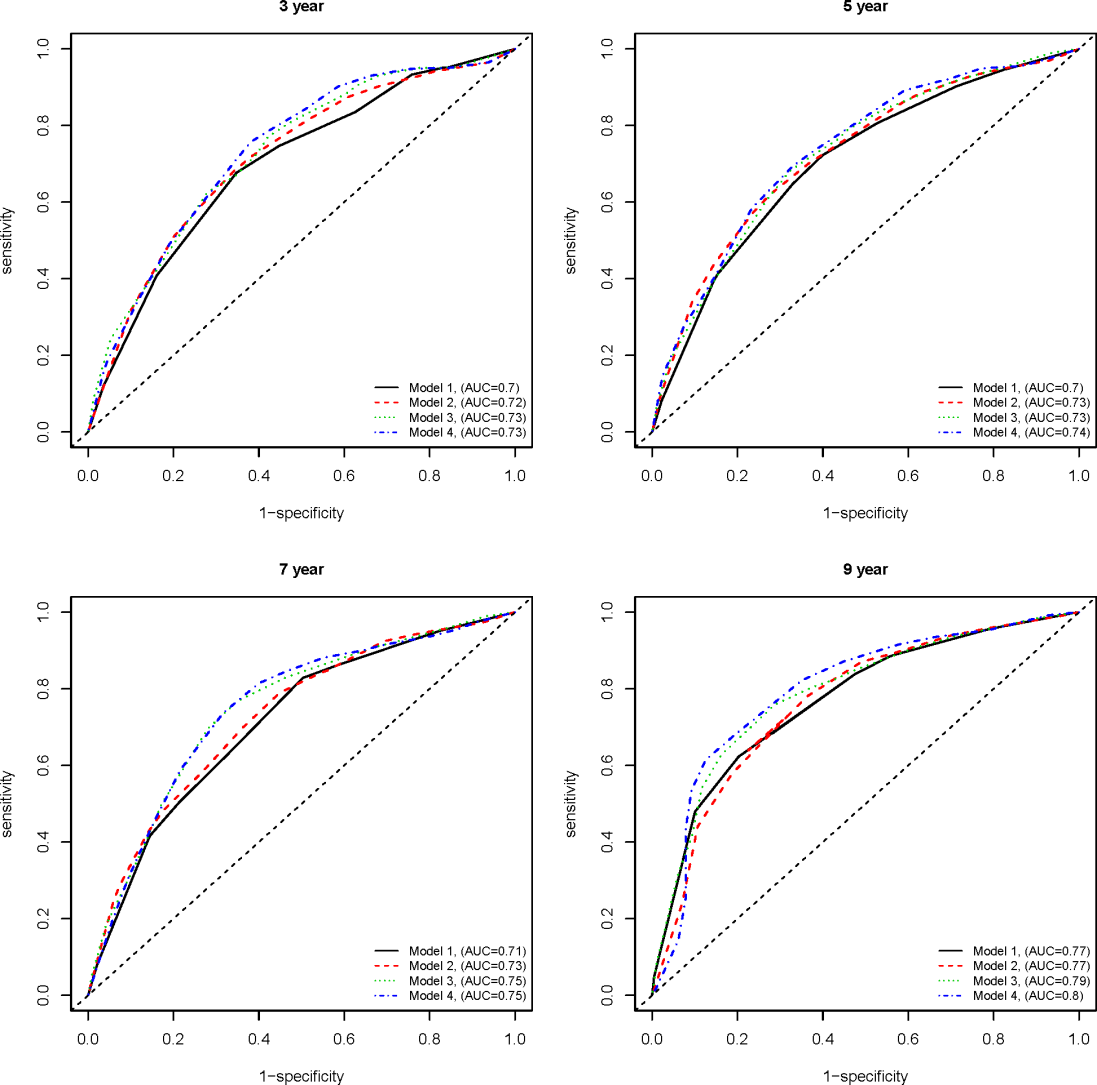
Time-dependent ROC curves at 3-, 5-, 7-, and 9-year follow-up times. A slight but consistent improvement can be seen comparing models containing the either or both of the polymorphisms to Model 1 at different time-points after diagnosis. Model 1) significant baseline characteristics only, Model 2) rs861539 + significant baseline characteristics, Model 3) rs34743033 + significant baseline characteristics, and Model 4) rs861539 + rs34743033 + significant baseline characteristics. ROC: receiver operator characteristic, AUC: area under the ROC curve.

We then proceeded with time-dependent ROC analysis, performed under four multivariable models: Model 1) significant baseline characteristics only, Model 2) *XRCC3-*rs861539 + significant baseline characteristics, Model 3) *TYMS*-rs34743033 + significant baseline characteristics, and Model 4) *XRCC3-*rs861539 + *TYMS*-rs34743033 + significant baseline characteristics. We also considered four different endpoints: specifically 3-, 5-, 7-, and 9-years follow-up time. As shown in Fig 1, there was a slight improvement in the discriminatory accuracy of models containing either one or the both polymorphisms when compared to Model 1 containing only the baseline characteristics. The most substantial improvement in each time-point is observed in Model 4, containing both polymorphisms in addition to the baseline variables. This was consistent across each of the follow-up times considered. As the follow-up time increases, there is a slight increase in the discriminatory accuracy under each model (Model 1–4) mainly due to the significant association of some of the baseline characteristics with the long-term risk of metastasis (Tables 3 and 4 and S1 Table).

## Discussion

Identifying genetic variations that can distinguish between patients in terms of their long-term risk of experiencing metastasis and timing of metastasis is essential in dissecting the biology behind metastasis, developing targeted therapeutics, and implementing precision medicine. This study focused on this particular challenge and examined 26 polymorphisms using a mixture cure model in the MSI-L/MSS subtype of colorectal cancer. The mixture cure model enabled us to dissect the short- and long-term effects of genetic markers on time to metastasis. Our main finding is that specific genotypes of two common polymorphisms, *XRCC3-*Thr241Met and *TYMS*-5’ UTR VNTR, were significantly associated with early metastasis in colorectal cancer. Patients with these genotypes were more likely to experience metastasis in a short time, but the long-term risk of metastasis was not significantly different in the patient subgroups. Additionally, time-dependent ROC curve analysis indicated these polymorphisms slightly increased the discriminatory accuracy of the model at different time points considered (Fig 1). The consistent increase in AUC obtained in the models containing these polymorphisms in addition to the significant baseline characteristics shows the overall improvement in our ability to discriminate between patients who will be free of metastasis and those who are susceptible to metastasis irrespective of follow-up time points. The results of this study therefore strongly suggest a potential importance of the *XRCC3-*Thr241Met and *TYMS*-VNTR polymorphisms in relation to time-to-metastasis in colorectal cancer.

XRCC3 is a DNA repair protein and key component of homologous recombination [23]. The *XRCC3* polymorphism investigated in this study is a nonsynonymous substitution resulting in an amino acid change from threonine to methionine at position 241 (Thr241Met). Previously, the variant protein has been shown to be associated with DNA adduct levels and increased risk of tetraploidy, linking this polymorphism to cancer [24,25]. A number of studies have looked at its association with clinical outcomes, including in colorectal cancer patients [15,26–30]. Among them, one study [27] associated the genotypes containing the variant allele to lower overall survival times in Asian patients. In our study these genotypes predicted early time-to-metastasis (Table 3, S3 Fig). This suggests that the *XRCC3* Thr241Met polymorphism deserves further functional characterization, particularly to examine whether it is biologically linked to metastasis.

*TYMS* polymorphisms are among the most studied variants in colorectal cancer due to the function of the encoded protein. *TYMS* codes for thymidylate synthase (TYMS), an enzyme that is involved in nucleotide synthesis. This enzyme can be inhibited by 5-FU, a chemotherapeutic agent commonly used in treatment of colorectal cancer [31]. In this study, we examined two *TYMS* polymorphisms; the 28 bp variable number of tandem repeat at the 5’ UTR and a 6 bp indel at the 3’ UTR. Our analysis identified that one of these variants was associated with outcome in colorectal cancer; specifically the homozygosity for the 3R allele of the 5’ UTR VNTR predicted early time-to-metastasis compared to 2R/3R and 2R/2R genotypes (Table 4, S3 Fig). This association can be biologically interesting as compared to the 2R genotype, the 3R genotype was previously associated with increased TYMS protein levels in colorectal/gastrointestinal tumor tissues [32,33]. Additionally, a systematic review and meta-analysis performed in 2004 concluded that colorectal tumors expressing higher TYMS protein levels appear to predict unfavorable prognosis when compared to tumors expressing low levels of TYMS [34]. However, despite being analyzed in multiple studies, the *TYMS* VNTR genotype–prognosis link in colorectal cancer remains inconclusive [35–40]. To our knowledge, only two previous studies [35,36] identified, similar to ours, significant associations of the 3R allele with outcomes in colorectal cancer; the 3R containing genotypes were associated with shorter time-to-progression in the study by Martinez-Balibrea et al. [35], and an increased risk of distant metastasis in the study by Gosens et al. [36]. In our study, patients with the 3R/3R genotype that experienced metastasis did so within the first 6–7 years after diagnosis (S3 Fig). After this time-point, patients with this genotype did not experience metastasis, despite the long-term follow-up for many patients. This suggests that if metastasis occurs in patients with this genotype, it is likely to be in a relatively short time after diagnosis. Thus, in the light of our and others findings, it is plausible to suggest that the 3R VNTR genotype of *TYMS* could have a direct biological role in accelerating disease progression/metastasis in colorectal cancer. Further experimental studies are needed to test this hypothesis.

The main strengths and limitations of this study can be summarized as follows: we recognize that our results need to be verified in other cohorts; the number of polymorphisms investigated in this study is relatively small; and this study focuses exclusively on patients from one ethnicity (Caucasian). Strengths include examining a well described patient cohort with long follow-up times, having a specifically defined end point (i.e., metastasis), focusing on a homogenous patient cohort by considering the MSI-L/MSS subtype of colorectal cancer, and application of the mixture cure modeling to adequately address our research questions.

In conclusion, we have identified two polymorphisms, *XRCC3-*Thr241Met and *TYMS* 5’ UTR VNTR, whose specific genotypes are significantly associated with early metastasis in colorectal cancer. These results, once replicated in other patient cohorts, could provide further insight into the variability of colorectal patient prognosis and provide potential biomarkers for early metastasis. Identifying such biomarkers that have time-varying effect on the risk of experiencing metastasis could be used in clinical management of patients by giving more appropriate treatment and surveillance decisions for different time periods.

## Supporting information

S1 Fig Kaplan-Meier survival functions stratified according to MSI status for the stage I-III patients. MSI: microsatellite instability; MSI-H: MSI-high; MSI-L: MSI-low; MSS: microsatellite stable.
(TIF)

S2 Fig Kaplan-Meier survival functions stratified according to the significant baseline characteristics. (TIF)

S3 Fig **(A) Kaplan-Meier survival functions stratified according to the *XRCC3* Thr241Met polymorphism or *TYMS* 5’-UTR VNTR polymorphism and (B) their corresponding conditional survival function estimates for the patients susceptible to metastasis.** Under the assumptions of the mixture cure model, the population is viewed as a mixture of susceptible and non-susceptible individuals to metastasis, where susceptible refers to patients who will experience metastasis and non-susceptible individuals are long-term metastasis-free survivors who are viewed as (statistically) cured. The upper plots in (A) show the Kaplan-Meier estimates of the survival curves of time-to-metastasis (*T*) for each genotype category considered (i.e., Kaplan-Meier estimates of the survival function *S*(*t*|*x*) = *Pr*(*T* > *t*|*x*) where *x* denotes the covariate (genotype category of the corresponding polymorphism)). On the other hand, the lower plots in (B) show the estimated conditional survival curves for the susceptible group under each *x* level (i.e., Kaplan-Meier estimates of *S*_0_(*t*|*x*) = *Pr*(*T* > *t* | *susceptible group*, *x*) which is the probability that the susceptible person will survive beyond a specified time *t* without metastasis). Hence, in the upper plots the probability of survival is for the population under consideration including both susceptible and non-susceptible individuals, but the lower plots are for the survival function of time-to-metastasis in the group of susceptible individuals. The conditional survival curves for the susceptible group in the lower plots are obtained from the mixture cure model *S*(*t*|*x*) = *Pr*(*T* > *t*|*x*) = *p*(*x*) + (1 − *p*(*x*))*S*_0_(*t*/*x*) where *p*(*x*) denotes the probability of being long-term metastasis-free survivor and thus 1 − *p*(*x*) is the probability of being susceptible to metastasis. Hence, the curves in the lower plots were obtained by plugging the Kaplan-Meier estimates of *S*(*t*|*x*) and *p*(*x*) in *S*_0_(*t*|*x*) = (*S*(*t*|*x*) − *p*(*x*))/(1 − *p*(*x*)).
(TIF)

S1 Table Multivariable mixture cure model results including all significant baseline characteristics identified: Tumor location, histologic grade, *BRAF* mutation status, 5-FU treatment status, and disease stage (n = 367 patients). HR: hazard ratio for time to metastasis among susceptible group. HR compares metastasis rate in subgroup *a* with that in subgroup *b* among those who are susceptible to metastasis. OR: odds ratio for metastasis (i.e., probability of being in susceptible group). OR compares metastasis proportion in subgroup *a* with that in subgroup *b*. CI: confidence interval; diff.: differentiated; 5-FU: 5- fluorouracil.
(DOCX)

S2 Table Multivariable mixture cure model results for each polymorphism examined in this study. Each row represents a separate fit and is adjusted for location, grade, *BRAF* mutation status, 5-FU treatment status, and disease stage. HR: hazard ratio for time to metastasis among susceptible group. HR compares metastasis rate in subgroup *a* with that in subgroup *b* among those who are susceptible to metastasis. OR: odds ratio for metastasis (i.e., probability of being in susceptible group). OR compares metastasis proportion in subgroup *a* with that in subgroup *b*. CI: confidence interval. *There were 3 patients with a 4R allele in *TYMS* rs34743033; this allele was treated like 3R during the analyses.
(DOCX)
