# Supplementary figures and images for: XRCC3 Thr241Met and TYMS variable number tandem repeat polymorphisms are associated with time-to-metastasis in colorectal cancer
Source: PLoS One. 2018 Feb 2;13(2):e0192316. doi: 10.1371/journal.pone.0192316 (PMC5796722; doi:10.1371/journal.pone.0192316)

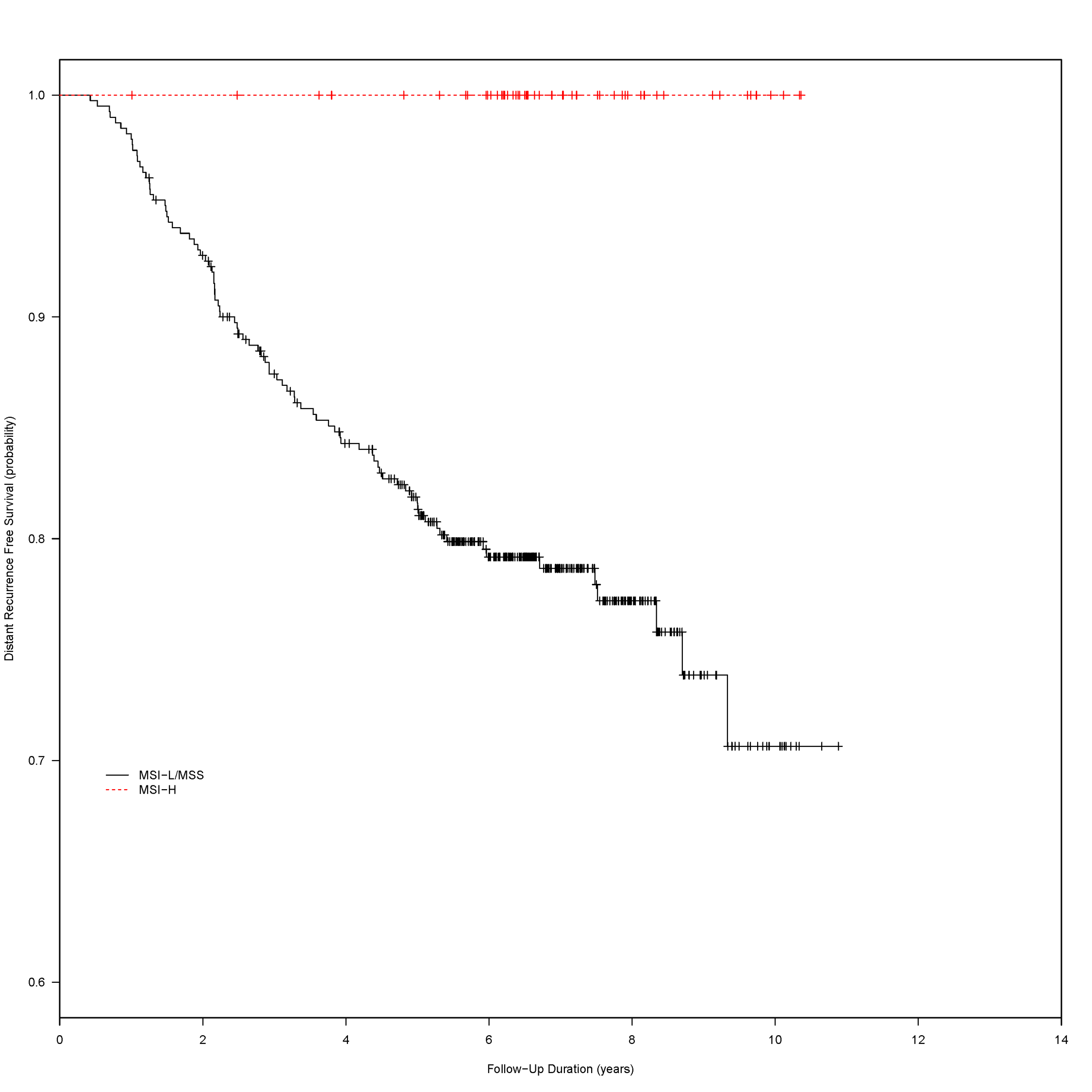

Supplement: S1 Fig — MSI: microsatellite instability; MSI-H: MSI-high; MSI-L: MSI-low; MSS: microsatellite stable. (TIF) [file pone.0192316.s001.tif]

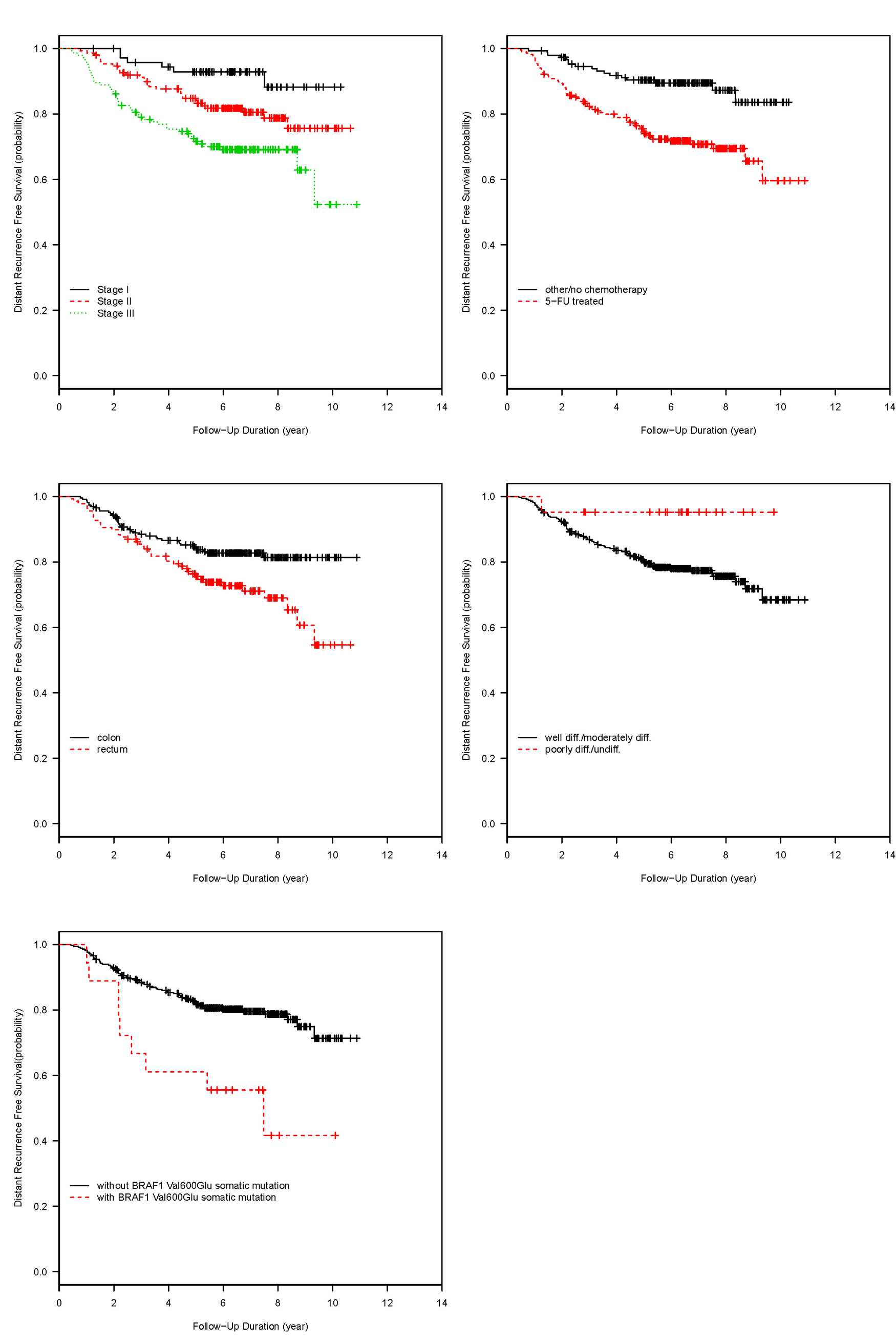

Supplement: S2 Fig — (TIF) [file pone.0192316.s002.tif]

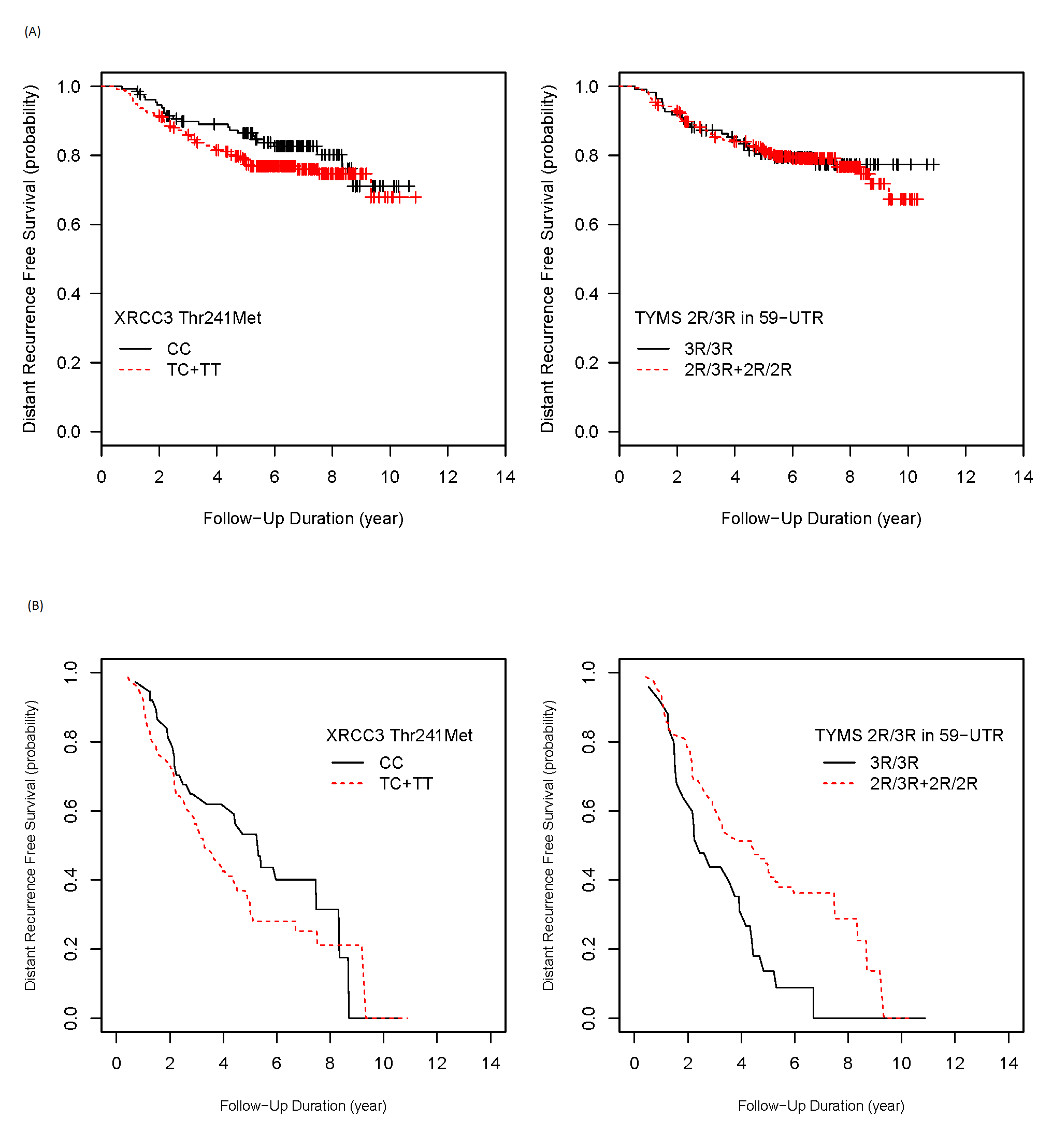

Supplement: S3 Fig — (A) Kaplan-Meier survival functions stratified according to the XRCC3 Thr241Met polymorphism or TYMS 5’-UTR VNTR polymorphism and (B) their corresponding conditional survival function estimates for the patients susceptible to metastasis. Under the assumptions of the mixture cure model, the population is viewed as a mixture of susceptible and non-susceptible individuals to metastasis, where susceptible refers to patients who will experience metastasis and non-susceptible individuals are long-term metastasis-free survivors who are viewed as (statistically) cured. The upper plots in (A) show the Kaplan-Meier estimates of the survival curves of time-to-metastasis (T) for each genotype category considered (i.e., Kaplan-Meier estimates of the survival function S(t|x) = Pr(T > t|x) where x denotes the covariate (genotype category of the corresponding polymorphism)). On the other hand, the lower plots in (B) show the estimated conditional survival curves for the susceptible group under each x level (i.e., Kaplan-Meier estimates of S0(t|x) = Pr(T > t | susceptible group, x) which is the probability that the susceptible person will survive beyond a specified time t without metastasis). Hence, in the upper plots the probability of survival is for the population under consideration including both susceptible and non-susceptible individuals, but the lower plots are for the survival function of time-to-metastasis in the group of susceptible individuals. The conditional survival curves for the susceptible group in the lower plots are obtained from the mixture cure model S(t|x) = Pr(T > t|x) = p(x) + (1 − p(x))S0(t/x) where p(x) denotes the probability of being long-term metastasis-free survivor and thus 1 − p(x) is the probability of being susceptible to metastasis. Hence, the curves in the lower plots were obtained by plugging the Kaplan-Meier estimates of S(t|x) and p(x) in S0(t|x) = (S(t|x) − p(x))/(1 − p(x)). (TIF) [file pone.0192316.s003.tif]
