# Supplementary material for: XRCC3 Thr241Met and TYMS variable number tandem repeat polymorphisms are associated with time-to-metastasis in colorectal cancer
Source: PLoS One. 2018 Feb 2;13(2):e0192316. doi: 10.1371/journal.pone.0192316 (PMC5796722; doi:10.1371/journal.pone.0192316)
Supplement: S1 Table — HR: hazard ratio for time to metastasis among susceptible group. HR compares metastasis rate in subgroup a with that in subgroup b among those who are susceptible to metastasis. OR: odds ratio for metastasis (i.e., probability of being in susceptible group). OR compares metastasis proportion in subgroup a with that in subgroup b. CI: confidence interval; diff.: differentiated; 5-FU: 5- fluorouracil. (DOCX) [file pone.0192316.s004.docx]

| **S1 Table. Multivariable mixture cure model results including all significant baseline characteristics identified: tumor location, histologic grade, *BRAF* mutation status, 5-FU treatment status, and disease stage (n=367 patients).** | | | | | | | |
| --- | --- | --- | --- | --- | --- | --- | --- |
|  | **Time-to-metastasis among susceptible patients** | | |  | **Long-term risk of metastasis** | | |
| **Prognostic Factor Characteristic (*a* vs. *b*)** | **HR** | **95% CI** | **p-value** |  | **OR** | **95% CI** | **p-value** |
| Location (Rectum vs. Colon) | 0.32 | (0.13, 0.78) | 0.013 |  | 7.20 | (1.54, 33.58) | 0.012 |
| Grade (poorly diff./undiff.  vs. well/moderately diff.) | 8.59 | (0.73, 100.40) | 0.086 |  | 0.04 | (0.00, 0.53) | 0.014 |
| *BRAF* Val600Glu Mutation Status  (Present vs. Absent) | 1.23 | (0.46, 3.30) | 0.682 |  | 4.70 | (1.41, 15.68) | 0.012 |
| 5-FU Treatment (Yes vs. No) | 0.29 | (0.10, 0.88) | 0.029 |  | 4.27 | (0.92, 19.83) | 0.064 |
| Stage II vs. Stage I | 2.28 | (0.19, 26.72) | 0.512 |  | 1.65 | (0.17, 15.56) | 0.664 |
| Stage III vs. Stage I | 9.72 | (0.75, 125.60) | 0.082 |  | 0.79 | (0.05, 13.24) | 0.868 |

HR: hazard ratio for time to metastasis among susceptible group. HR compares metastasis rate in subgroup *a* with that

in subgroup *b* among those who are susceptible to metastasis.

OR: odds ratio for metastasis (i.e., probability of being in susceptible group). OR compares metastasis proportion in

subgroup *a* with that in subgroup *b*.

CI: confidence interval; diff.: differentiated; 5-FU: 5- fluorouracil.
