# Supplementary material for: XRCC3 Thr241Met and TYMS variable number tandem repeat polymorphisms are associated with time-to-metastasis in colorectal cancer
Source: PLoS One. 2018 Feb 2;13(2):e0192316. doi: 10.1371/journal.pone.0192316 (PMC5796722; doi:10.1371/journal.pone.0192316)
Supplement: S2 Table — Each row represents a separate fit and is adjusted for location, grade, BRAF mutation status, 5-FU treatment status, and disease stage. HR: hazard ratio for time to metastasis among susceptible group. HR compares metastasis rate in subgroup a with that in subgroup b among those who are susceptible to metastasis. OR: odds ratio for metastasis (i.e., probability of being in susceptible group). OR compares metastasis proportion in subgroup a with that in subgroup b. CI: confidence interval. *There were 3 patients with a 4R allele in TYMS rs34743033; this allele was treated like 3R during the analyses. (DOCX) [file pone.0192316.s005.docx]

| **S2 Table. Multivariable mixture cure model results for each polymorphism examined in this study.** | | | | | | | |
| --- | --- | --- | --- | --- | --- | --- | --- |
|  | **Time-to-metastasis among susceptible patients** | | |  | **Long-term risk of metastasis** | | |
| **Polymorphism (*a* vs. *b*)** | **HR** | **95% CI** | **p-value** |  | **OR** | **95% CI** | **p-value** |
| *ERCC2* rs13181 (GG+GT vs. TT) | 1.01 | (0.55, 1.86) | 0.970 |  | 0.55 | (0.24, 1.30) | 0.173 |
| *GSTP1* rs1695 (GG+GA vs. AA) | 1.44 | (0.74, 2.80) | 0.288 |  | 0.72 | (0.34, 1.54) | 0.404 |
| *MTHFR* rs1801131 (CC+CA vs. AA) | 1.22 | (0.62, 2.43) | 0.565 |  | 0.97 | (0.42, 2.22) | 0.944 |
| *VEGFA* rs2010963 (CC+GC vs. GG) | 0.79 | (0.40, 1.55) | 0.490 |  | 1.07 | (0.45, 2.55) | 0.879 |
| *XRCC1* rs25487 (AA+AG vs. GG) | 0.79 | (0.45, 1.38) | 0.407 |  | 1.93 | (0.80, 4.63) | 0.141 |
| *ERCC5* rs1047768 (TT+TC vs. CC) | 1.52 | (0.63, 3.65) | 0.348 |  | 0.92 | (0.25, 3.31) | 0.893 |
| *OGG1* rs1052133 (GG+GC vs. CC) | 1.95 | (0.86, 4.42) | 0.109 |  | 1.07 | (0.50, 2.29) | 0.862 |
| *ERCC1* rs11615 (CC+TC vs. TT) | 0.66 | (0.30, 1.45) | 0.305 |  | 1.77 | (0.83, 3.79) | 0.139 |
| *TYMS* rs16430 (-/- + 6 bp/- vs. 6 bp/6 bp) | 1.06 | (0.45, 2.51) | 0.892 |  | 0.89 | (0.36, 2.21) | 0.798 |
| *MLH1* rs1799977 (GG+GA vs. AA) | 0.60 | (0.26, 1.39) | 0.237 |  | 1.57 | (0.76, 3.26) | 0.226 |
| *TYMS* rs34743033 (2R/2R + 2R/3R vs. 3R/3R*) | 0.40 | (0.20, 0.80) | 0.009 |  | 2.41 | (0.85, 6.82) | 0.096 |
| *FAS* rs1800682 (CC+TC vs. TT) | 0.88 | (0.41, 1.87) | 0.735 |  | 0.78 | (0.35, 1.76) | 0.554 |
| *IL6* rs1800795 (CC+GC vs. GG) | 1.33 | (0.62, 2.87) | 0.462 |  | 1.01 | (0.38, 2.70) | 0.980 |
| *EGFR* rs2227983 (AA+GA vs. GG) | 0.90 | (0.45, 1.79) | 0.757 |  | 0.91 | (0.42, 1.97) | 0.809 |
| *DCC* rs2229080 (GG+CG vs. CC) | 0.77 | (0.38, 1.56) | 0.468 |  | 0.73 | (0.33, 1.63) | 0.446 |
| *VEGFA* rs3025039 (TT+CT vs. CC) | 0.68 | (0.27, 1.68) | 0.402 |  | 0.92 | (0.34, 2.53) | 0.875 |
| *FGFR4* rs351855 (TT+CT vs. CC) | 1.47 | (0.76, 2.82) | 0.249 |  | 0.83 | (0.39, 1.77) | 0.621 |
| *XRCC3* rs861539 (TT+TC vs. CC) | 2.06 | (1.03, 4.13) | 0.042 |  | 1.05 | (0.46, 2.40) | 0.911 |
| *CCND1* rs9344 (AA+GA vs. GG) | 1.20 | (0.54, 2.63) | 0.655 |  | 1.31 | (0.56, 3.09) | 0.536 |
| *EXO1* rs9350 (TT+CT vs. CC) | 1.75 | (0.88, 3.48) | 0.112 |  | 0.52 | (0.17, 1.60) | 0.255 |
| *SERPINE1* rs1799889 (GG + G/- vs. -/-) | 0.71 | (0.28, 1.76) | 0.455 |  | 1.73 | (0.70, 4.23) | 0.233 |
| *MMP1* rs1799750 (GG + G/- vs. -/-) | 1.23 | (0.59, 2.57) | 0.584 |  | 0.87 | (0.31, 2.46) | 0.798 |
| *GSTT1* Gene deletion (deleted vs. present) | 0.47 | (0.11, 1.93) | 0.293 |  | 0.37 | (0.09, 1.42) | 0.147 |
| *GSTM1* Gene deletion (deleted vs. present) | 1.19 | (0.44, 3.20) | 0.729 |  | 1.99 | (0.82, 4.83) | 0.129 |
| *MMP2* rs243865 (TT+CT vs. CC) | 0.49 | (0.23, 1.04) | 0.064 |  | 1.52 | (0.60, 3.85) | 0.381 |
| *MTHFR* rs1801133 (TT+TC vs. CC) | 0.48 | (0.23, 1.00) | 0.051 |  | 1.16 | (0.53, 2.55) | 0.704 |

Each row represents a separate fit and is adjusted for location, grade, *BRAF* mutation status, 5-FU treatment status, and disease stage.

HR: hazard ratio for time to metastasis among susceptible group. HR compares metastasis rate in subgroup *a* with that in subgroup *b* among those who are susceptible to metastasis.

OR: odds ratio for metastasis (i.e., probability of being in susceptible group). OR compares metastasis proportion in subgroup *a* with that in subgroup *b*.

CI: confidence interval.

*There were 3 patients with a 4R allele in *TYMS* rs34743033; this allele was treated like 3R during the analyses.
